# Attenuation of immobilization stress-induced hypertension by temperature-controllable warm needle acupuncture in rats and the peripheral neural mechanisms

**DOI:** 10.3389/fneur.2023.1168012

**Published:** 2023-06-13

**Authors:** Se Kyun Bang, Suchan Chang, Su Yeon Seo, Suk-Yun Kang, Seong Jin Cho, Kwang-Ho Choi, Xing Juping, Hee Young Kim, Yeonhee Ryu

**Affiliations:** ^1^Department of Korean Medicine Science Research Division, Korea Institute of Oriental Medicine, Daejeon, Republic of Korea; ^2^Department of Korean convergence Medical Science, University of Science and Technology, Daejeon, Republic of Korea; ^3^Department of Physiology, College of Korean Medicine, Daegu Haany University, Daegu, Republic of Korea; ^4^Department of Physiology, Yonsei University College of Medicine, Seoul, Republic of Korea

**Keywords:** stress, hypertension, warm needle acupuncture, peripheral sensory nerve, TRPV1

## Abstract

**Introduction:**

We and others have shown that electrical stimulation of the PC-6 acupoint over the wrist relieves hypertension by stimulating afferent sensory nerve fibers and activating the central endogenous opioid system. Warm needle acupuncture has long been utilized to treat various diseases in clinics.

**Methods:**

Here, we developed a temperature-controllable warm needle acupuncture instrument (WAI) and investigated the peripheral mechanism underlying the effect of warm needle acupuncture at PC-6 on hypertension in a rat model of immobilization stress-induced hypertension.

**Results:**

Stimulation with our newly developed WAI and traditional warm needle acupuncture attenuated hypertension development. Such effects were reproduced by capsaicin (a TRPV1 agonist) injection into PC-6 or WAI stimulation at 48°C. In contrast, PC-6 pretreatment with the TRPV1 antagonist capsazepine blocked the antihypertensive effect of WAI stimulation at PC-6. WAI stimulation at PC-6 increased the number of dorsal root ganglia double-stained with TRPV1 and CGRP. QX-314 and capsaicin perineural injection into the median nerve for chemical ablation of small afferent nerve fibers (C-fibers) prevented the antihypertensive effect of WAI stimulation at PC-6. Additionally, PC-6 pretreatment with RTX ablated the antihypertensive effect of WAI stimulation.

**Conclusion:**

These findings suggest that warm needle acupuncture at PC-6 activates C-fiber of median nerve and the peripheral TRPV1 receptors to attenuate the development of immobilization stress-induced hypertension in rats.

## 1. Introduction

Acupuncture is a traditional medicine that has been used to treat various diseases for thousands of years. Depending on the disease, acupuncture stimulates specific points on the skin, which are called acupuncture points or acupoints (1, 2). Various studies are being conducted to identify anatomically invisible acupoints. Li et al. reported that many acupoints are located in a place with a high density of sensory nerve endings (3). Kim et al. have suggested that some acupoints are identical to neurogenic inflammatory spots on the skin, which are produced by the activation of somatic afferents in abnormal conditions of visceral organs (4). For acupuncture treatment, there are various methods for stimulating acupoints, including physical stimulation by needle insertion, heat stimulation using moxa and warm needle acupuncture, electrical stimulation, laser stimulation, magnetic stimulation, and ultrasonic stimulation (5, 6). As one of the commonly used methods in acupuncture medicine, Based on previous publications (7, 8), warm needle acupuncture can be defined as a combination of acupuncture and thermal therapy (with heat sources like moxa, laser or heating devices). The temperature of needle following heating revealed large variations ranging from 30 to 66°C. In traditional acupuncture theory, the use of warm needle acupuncture is described to transmit moxa heat deeply into the body by way of acupuncture points, promoting the circulation of the Qi-Blood and activating the meridian system (9). Warm needle acupuncture has been reported to be effective in the treatment of various diseases and disorders, such as rheumatoid diseases (10), musculoskeletal disorders (11, 12), and insomnia (13), although the underlying mechanisms remain to be identified.

Several studies have demonstrated that the stimulation of Nei-guan (PC-6), an acupoint over the wrist, is most effective in lowering blood pressure and improving cardiovascular diseases (14). Our clinical trials showed that PC-6 electrical stimulation induces a decrease in blood pressure through the activation of the C-fibers of the median nerve (15). In addition, animal studies have shown that electrical stimulation of PC-6 induces endogenous opioid release in the rostral ventrolateral medulla (rVLM) and reduces the excitatory responses of cardiac afferent neurons to produce antihypertensive effects in a rat model of immobilization stress-induced hypertension (IMH) (16). Thermal stimulation of acupoints by warm needle acupuncture has been used in the treatment of cardiovascular diseases (17, 18), but the peripheral mechanism underlying the effect of warm needle acupuncture on cardiovascular diseases has not been identified.

It is generally accepted that acupuncture signals are initiated by several peripheral sensory receptors that detect mechanical stimulation, temperature and nociception (19). If thermal stimulation at acupoints is applied, the thermal stimulation may be transformed as electrical signals through transient receptor potential vanilloid (TRPV) channels (20), which are well-known temperature sensors (21). According to their structure and function, TRPV channels are classified into the following four groups: TRPV1, TRPV2, TRPV3 and TRPV4. TRPV channels on a primary sensory neuron are mainly expressed in nonmyelinated C-fibers, myelinated A-fibers and DRG neurons (22). In particular, TRPV1 is a nonselective cation channel with high calcium permeability that reacts with capsaicin, protons (pH < 5.9), and noxious heat (43–52°C) (23). As warm needle acupuncture utilizes heat over 40°C, TRPV1 may be closely associated with the effects of warm needle acupuncture.

To explore the peripheral mechanism underlying the effects of warm needle acupuncture on immobilization stress-induced hypertension in rats, we used our newly constructed device (Warm needle acupuncture instrument; WAI) to overcome the variability intensity and duration of thermal stimulation typically inherent in traditional warm needle acupuncture. After that, we tested the antihypertensive effect of warm needle acupuncture, and explored whether the effect of warm needle acupuncture was associated with the TRPV1 channel.

## 2. Materials and methods

### 2.1. Animals

Male Sprague–Dawley rats (weight 320 ~ 350 g, Samtako Bio, Osan, Korea) were housed at constant humidity (50 ~ 60%) and temperature (22 ± 2°C) with free access to food and water under a 12-h/12-h light/dark cycles. All experiments were approved by the Institutional Animal Care and Use Committee of the Korea Institute of Oriental Medicine with approval number #21–020 (Daejeon, Korea).

### 2.2. Chemicals

Capsazepine (20 nmol/30 μL in 100% dimethyl sulfoxide (DMSO); 30 μL/loci, Sigma–Aldrich, St. Louis, MO, United States), resiniferatoxin (RTX; 250 ng/30 μL in 95% ethanol; 30 μL/loci, Alomone Labs, Jerusalem, Israel), QX-314 (dissolved in saline; 50 μL/loci, lidocaine N-ethyl bromide; Tocris, Bristol, United Kingdom), and capsaicin (dissolved in 10% ethanol and 5% Tween 80; 50 μL/loci, Sigma–Aldrich, St. Louis, MO, United States) were used. Isolectin GS-IB4 antibody, Alexa Fluor 488 conjugate (IB4, I21411, Invitrogen, Gland Island, NY, United States), human calcitonin gene-related peptide antibody (CGRP; ab47027, Abcam, Cambridge, MA, United States), neurofilament 200 antibody (NF200, N4142, Sigma–Aldrich, St. Louis, MO, United States), TRPV1 antibody (VR1, SC-398417, Santacruz biotechnology, Santacruz, United States), and PGP 9.5 antibody (ab8189, Abcam, Cambridge, United Kingdom) were used.

### 2.3. Development of the WAI and acupuncture treatment

**Figure 1:**
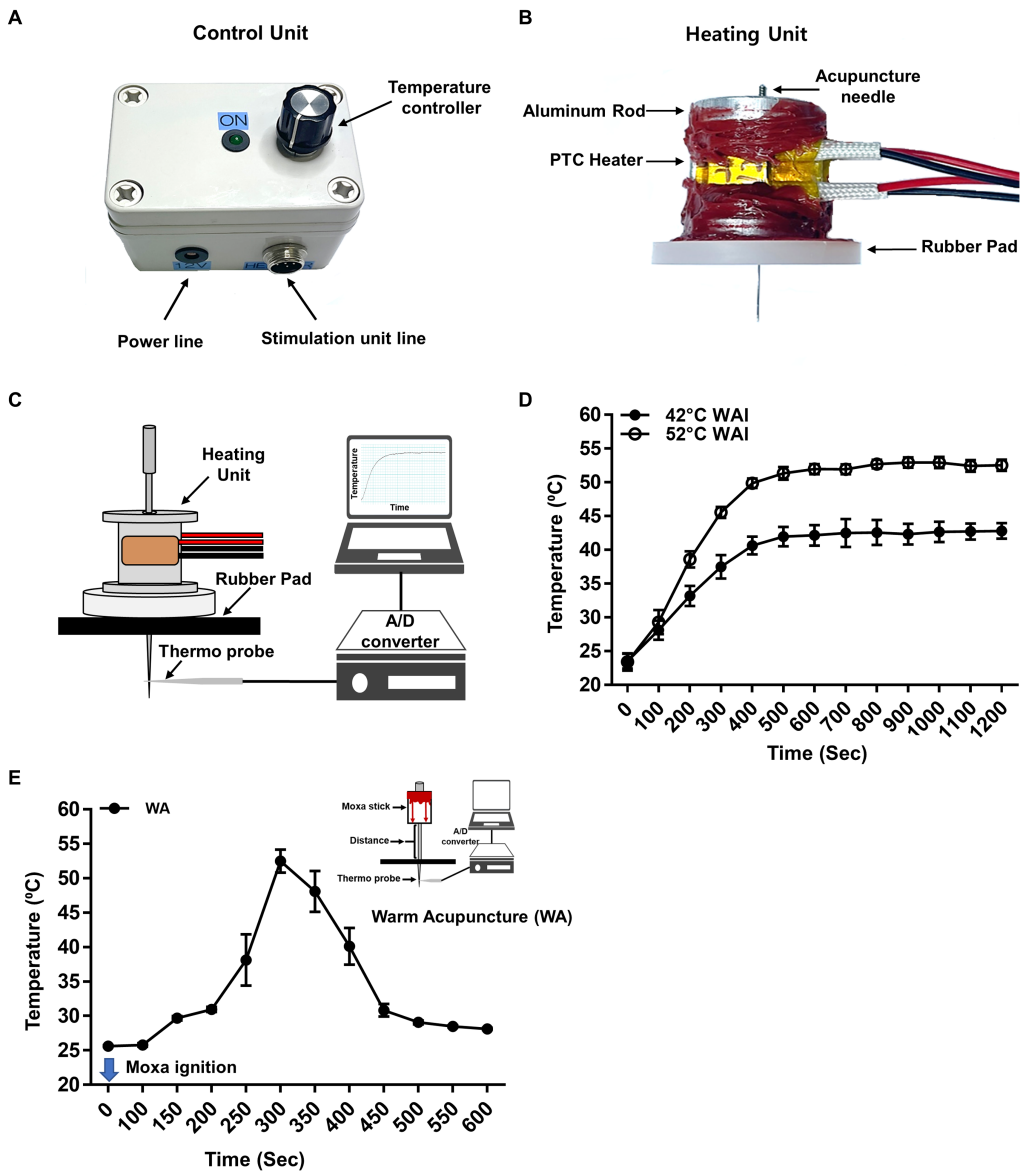
Warm needle acupuncture instrument (WAI). **(A)** Control unit. The WAI consists of a temperature controller and a heating unit. **(B)** Heating unit. Two positive temperature coefficient (PTC) heaters were fixed to an aluminum rod, and a rubber pad was attached to the bottom. The temperature of the heating unit was adjusted by the voltage of the control unit. **(C)** Schematics for temperature measurement at the tip of the acupuncture needle during WAI operation. The acupuncture needle was placed in a rubber pad through the heating unit, and a needle temperature probe was placed 0.3 mm below the needle. **(D)** The temperature delivered to the needle tip when the WAI was set to 42°C (*n* = 10) or 52°C (*n* = 10). **(E)** Temperature of needle tip during warm needle acupuncture (WA) (*n* = 10).

The WAI was developed to mimic traditional warm needle acupuncture. To quantitatively control the temperature of the inserted needle, the WAI (Figure 1A) consisted of a heating unit, a control unit, and a power supply. For the heating unit (Figure 1B), two positive temperature coefficient (PTC) ceramic heaters (12x8x3 mm, LxWxT, Gaoyi, China) were fixed to both sides of a custom-made aluminum rod, and an insulating rubber pad was attached to the bottom to prevent skin burns due to heat.

For acupuncture treatment, the rats were anesthetized with 3% isoflurane via a nose cone. Acupuncture needles were bilaterally inserted into the PC-6 acupuncture points at a depth of 2 mm from the skin. For the stable temperature of 42°C, 48°C, or 52°C, the WAI device was placed over the acupuncture needle after the device was heated for at least 10 min at the preset temperature. WAI stimulation was performed for 10 min, anesthesia was discontinued, and blood pressure was measured 10 min after the cessation of anesthesia. To see whether the WAI stimulation causes the morphological tissue damage, the tissues were taken out after 52°C warm needle acupuncture or sham operation (needle insertion but no heating). The tissues were postfixed in 4% paraformaldehyde and embedded in paraffin-blocks, sectioned at 5 μm-thickness, stained with Hematoxylin & Eosin (H&E) and examined under the bright-field microscope.

For traditional warm needle acupuncture (WA), a 15 mm moxa stick (Dongbang Medical Co, Korea) were placed on the handle of the acupuncture needles (diameter 0.20 mm, length 40 mm, Dongbang Medical Co, Korea), and the needles were bilaterally inserted 2 mm deep into PC-6. The moxa was allowed to burn fully for at least 10 min while rats were under anesthesia.

### 2.4. Measurement of the temperature delivered to the needle by the WAI or WA

The temperature delivered to the needle by the WAI or WA was measured by using our customized temperature measurement system. Acupuncture needles (0.10 mm in diameter, needle length of 10 mm, and handle length of 10 mm; Dongbang Medical Co, Korea) were inserted into the rubber pad to a depth of 2 mm. A thermocouple microprobe (NJ-07013, WPI, Sarasota, FL, United States) was then attached to the tip of the needle, and the temperature was measured for 20 min by using a digital temperature monitoring device (BAT-12, Physitemp, New Jersey, United States). For WA, a moxa stick (10 mm in diameter and 15 mm height; Dongbang Medical Co, Korea) was attached to the shaft of the acupuncture 2 cm away from the needle tip. Temperature was measured for 10 min after igniting the moxa. Data were analyzed using the Powerlab 4/30 acquisition system (Powerlab, AD Instruments, Inc., Colorado, United States).

### 2.5. Pharmacological blockade of TRPV1 receptors and small afferent nerve fibers

To block peripheral TRPV1 receptors, capsazepine (10 mM; 30 μL/loci, s.c.) was injected into the PC-6 acupuncture point over the wrist at a depth of 3 mm vertically from the skin surface using a 26-gauge Hamilton syringe (Reno, NV, United States) 15 min before WAI treatment. To block TRPV1 on small afferent nerve fibers (Aδ/C-fibers), RTX (250 ng/30 μL/loci, s.c) (24) was injected into the PC-6 acupoint 24 ~ 48 h prior to WAI stimulation, as previously described (25).

### 2.6. Pharmacological blockade of C-fibers in the median nerve

Pharmacological blockade of C-fibers in the median nerve was performed as previously described (26). A mixture of QX-314 and capsaicin, which selectively blocks sodium channels and inhibits excitability in nociceptors (27), was injected perineurally into the median nerve. In brief, while rats were under isoflurane anesthesia, approximately 0.5 cm of the skin on the inner side of the elbow was excised to expose the median nerve, and 50 μL of QX-314 (1%) and capsaicin (0.5 μg/μL) was administered to the perineural space of the median nerve. After skin suturing, ketoprofen (5 mg/kg SC) was administered near the surgical site. WAI stimulation was performed 1 h after injection.

### 2.7. Measurement of blood pressure and immobilization stress-induced hypertension (IMH)

Hypertension was induced by restraining the rats using cone-shaped plastic bags (length 185 mm, internal diameter 59 mm), as previously described (28), and systolic blood pressure was measured by using a noninvasive blood pressure measurement system. The animal was placed into a prewarmed chamber of 27 ~ 30°C for at least 30 min. A programmed electro-sphygmomanometer (Narco Bio-Systems, Inc., Houston, United States) was inflated and deflated automatically, and the tail cuff signals from the transducer were automatically collected using an IITC apparatus (Model 47, IITC, Inc., California, United States). Each blood pressure measurement was taken as an average of two readings every 10 min. Rats with systolic blood pressure of over 160 mmHg were defined as hypertensive (29).

### 2.8. Immunohistochemical analysis of dorsal root ganglia (DRGs) and skin

Immunohistochemical analysis of DRGs and skin was carried out as described previously (26, 30). In brief, 30 min after bilateral WAI stimulation of the rats, the C5-8 DRGs and wrist skin over the PC-6 acupoint (5× 5 × 5 mm) were removed after perfusion with phosphate-buffered saline (PBS) and 4% paraformaldehyde. The DRG samples were cryosectioned at a thickness of 10 μm. Skin samples were paraffin-embedded and sectioned at 5 μm thickness. The slides were mounted on poly-L-lysine-coated glass slides (Thermo Scientific, MA, United States). The slides were incubated with mouse anti-TRPV1 antibody (1:500, Santa Cruz Biotechnology), rabbit anti-calcitonin gene-related peptide (CGRP, 1:1000, Abcam), anti-lectin IB4 Alexa Fluor 488 conjugated to FITC (1:500, Invitrogen), rabbit anti-neurofilament 200 (NF200, 1:1000, Sigma–Aldrich) and mouse monoclonal PGP 9.5 antibody (1:1000; Abcam, Cambridge, United Kingdom; ab8189) overnight at 4°C, followed by incubation with secondary antibodies (1:200, Alexa Fluor 488 donkey anti-rabbit IgG antibody), Thermo Scientific, MA, United States, Product# A-21206; 1:200, Alexa Fluor^®^ 594 donkey anti-mouse IgG antibody, Thermo Scientific, Product# A-21203, donkey anti-mouse IgG Cy3 (1:500, 133,644, Invitrogen). The slides were washed and cover-slipped with Vectashield Hard Set mounting medium with DAPI (4′, 6-diamidino-2-phenylindole, blue, Vector, United States, Product# H-1500). All DRG and skin samples were taken from 3 ~ 5 sections from each animal. All images were taken with a fluorescence microscope (BX51; Olympus, Hamburg, Germany), and the value of positive pixels within a field area of 300 × 240 pixels was calculated with ImageJ (version 1.44d software National Institutes of Health, Bethesda, MD).

### 2.9. Data analysis

All data are shown as the mean ± SEM (standard error of the mean) and were analyzed by one- or two-way analysis of variance (ANOVA) followed by Tukey’s *post hoc* test or paired or unpaired t test, where appropriate. All statistics were calculated with GraphPad Prism 6 (GraphPad Software Incorporation, San Diego, United States). Differences were determined to be statistically significant when *p* values were < 0.05.

## 3. Results

### 3.1. Temperature measurement at the tip of the acupuncture needle during WAI operation and WA

A device was newly constructed for heating acupuncture needles to mimic traditional warm needle acupuncture and consisted of a control unit (Figure 1A) and a heating unit (Figure 1B). To ensure that the heat delivery to the needle at the preset temperature was stable, the temperature at the needle tip during heating was measured by using a temperature monitoring system combined with a thermal needle electrode (Figure 1C). After setting the WAI temperature at 52°C or 42°C, temperature measurements were made for 20 min. When the WAI was set to 52°C, the temperature at the needle tip started at 23.51°C ± 0.4524 and stably reached 52.69°C ± 0.3127 in 600 s. When the WAI was set to 42°C, the temperature at the needle tip started at 23.40°C ± 0.5252 and reached 42.13°C ± 0.6221 after 600 s (Figure 1D). These data showed that the WAI successfully led to the desired temperatures at the needle tip. On the other hand, the maximum temperature of needle tip during warm acupuncture (WA) was about 52°C 5 min after ignition (Figure 1E).

### 3.2. Effect of warm needle acupuncture at PC-6 on systolic blood pressure in an IMH rat model

**Figure 2:**
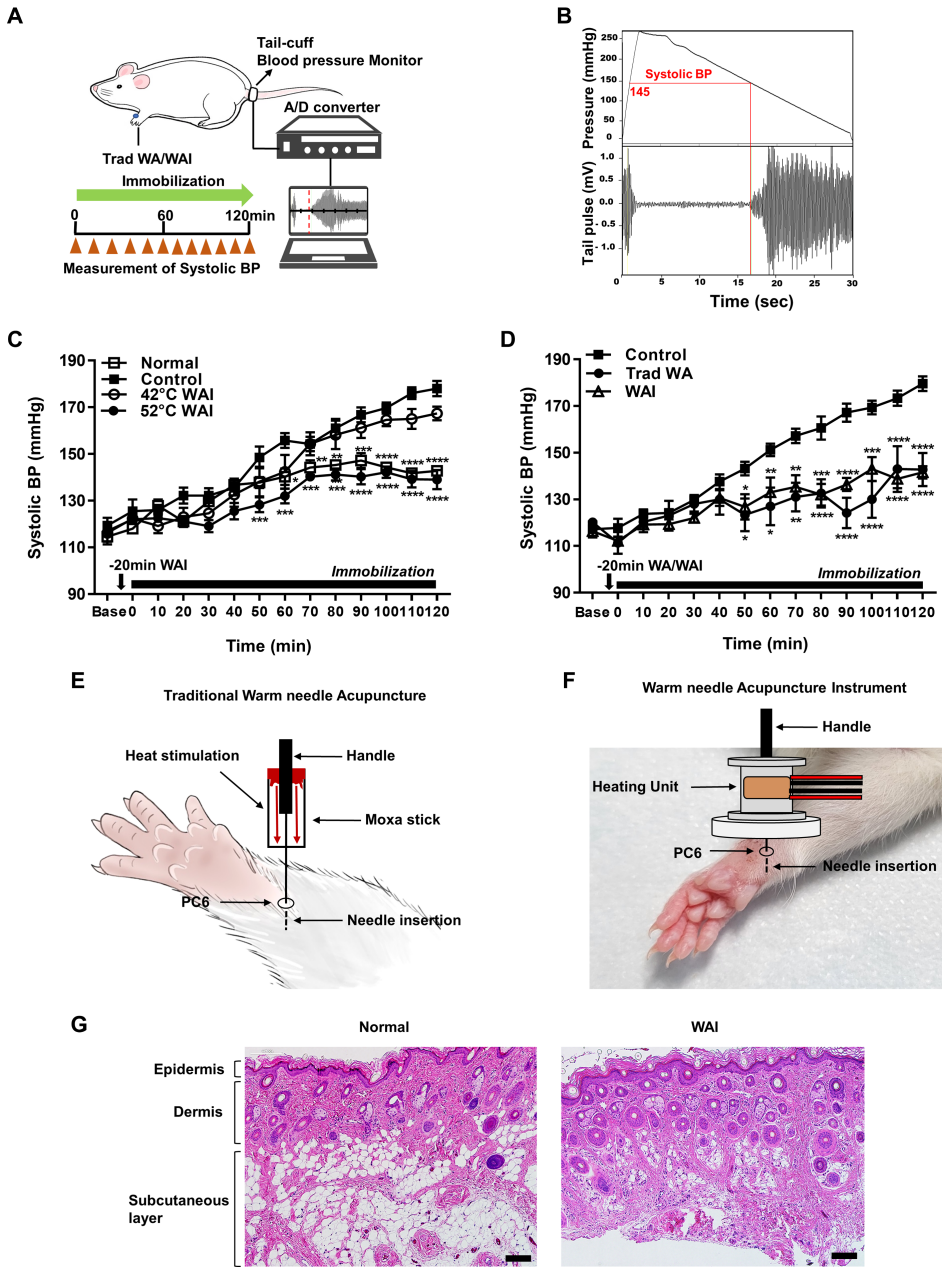
Effect of warm needle acupuncture at PC-6 on systolic blood pressure (BP) in immobilization stress-induced hypertension (IMH) rats. **(A)** Schematic showing the experimental procedure for the measurement of systolic BP in the rat model of IMH. **(B)** A representative example of the measurement of BP in a computer-controlled system. **(C)** Effect of warm needle acupuncture instrument (WAI) stimulation at 42°C (*n* = 6) or 52°C (*n* = 6) at PC-6 on the development of IMH in rats. Normal group (*n* = 6, unrestrained), ^****^p < 0.0001 vs. Control (*n* = 6, immobilization only); 42°C WAI, WAI stimulation of PC-6 at 42°C; 52°C WAI, WAI stimulation of PC-6 at 52°C. **(D)** Comparison of traditional warm needle acupuncture (Trad WA) and WAI stimulation at PC-6 on the development of hypertension in rats. ^****^p < 0.0001 vs. Control. 5 rats per group. **(E)** Schematic for traditional warm needle acupuncture at PC-6. **(F)** Schematic for the WAI at PC-6. **(G)** Representative H&E staining images of normal and 52°C WAI stimulation at PC-6. Scale bar = 100 μm.

To explore the effect of WAI stimulation at two different temperatures, 42 or 52°C, on hypertension, systolic blood pressure following WAI stimulation was measured in the tail of rats with IMH by using a noninvasive blood pressure monitoring system (Figures 2A,B). The blood pressure in rats with IMH significantly increased from 119.33 mmHg to 178 mmHg over the next couple of hours compared with the values before IMH or in the normal group (Figure 2C). When WAI stimulation at 42°C or 52°C was applied by the needle inserted into PC-6, the 52°C WAI group exhibited a significant attenuation of hypertension compared to the control group. However, WAI stimulation at 42°C failed to inhibit the development of IMH in rats (two-way ANOVA; group *F* (3,19) = 29.73, *p* < 0.0001; time *F* (13,247) = 87.28, *p* < 0.0001; interaction *F* (39,247) = 4.675, *p* < 0.0001).

To determine whether stimulation with our newly developed WAI (Figure 2F) was as effective as traditional warm needle acupuncture, the effects of WAI stimulation at 52°C on hypertension were compared with those of traditional warm needle acupuncture (Figure 2E). While immobilized rats showed the development of hypertension (Control), WAI stimulation as well as traditional warm needle acupuncture (Trad WA) at PC-6 significantly blocked the development of hypertension from 50 min after stimulation in rats with IMH compared to control rats (Figure 2D) (two-way repeated ANOVA; group *F* (2,168) = 71.72, *p* < 0.0001; time *F* (13,168) = 22.84, *p* < 0.0001; interaction *F* (26,168) = 3.383, *p* < 0.0001). Histological examination revealed that WAI stimulation at 52°C did not cause morphological tissue damages (Figure 2G).

### 3.3. Mediation of median nerve C-fibers in the antihypertensive effect of WAI stimulation at PC-6

**Figure 3:**
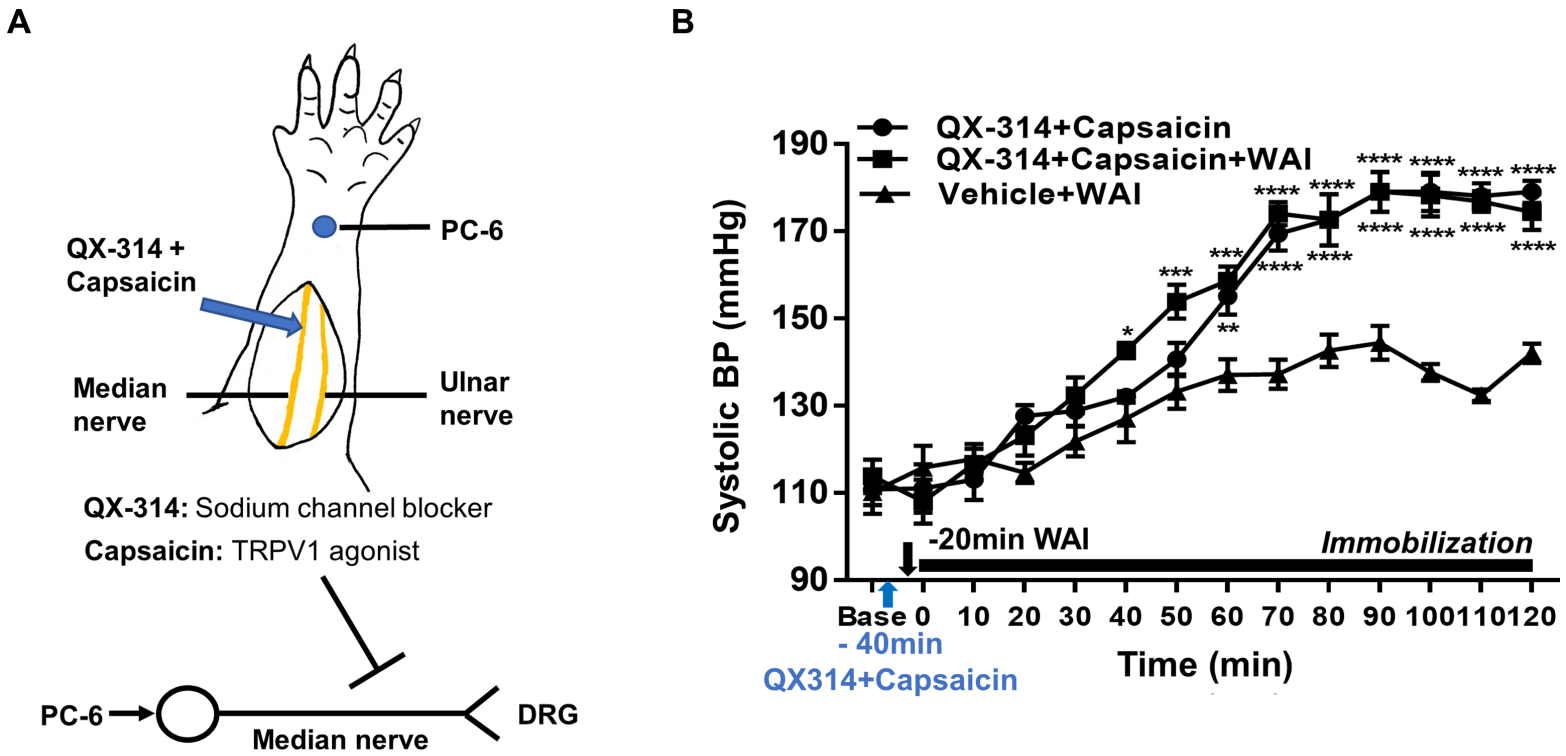
Effect of QX-314 and capsaicin preinjection into the median nerve on PC-6 warm needle acupuncture instrument (WAI) stimulation-induced inhibition of IMH in rats. **(A)** Schematic for blocking C-fibers by administering a mixture of QX-314 and capsaicin into the median nerve. **(B)** Effect of pretreatment with QX-314 and capsaicin on PC-6 WAI stimulation-induced the inhibition of hypertension. ^****^p < 0.0001 vs. Vehicle + WAI (*n* = 5). QX-314 + Capsaicin, QX-314 and capsaicin were injected into the median nerve (*n* = 5); QX-314 + Capsaicin+ WAI, QX-314 and capsaicin were injected into the median nerve prior to 52°C WAI stimulation at PC-6 (*n* = 5); Vehicle+ WAI (52°C), vehicle was injected into the median nerve prior to WAI stimulation (*n* = 5).

To explore the possible mediation of median nerve C-fibers in the antihypertensive effect of WAI stimulation at PC-6, a mixture of QX-314 and capsaicin, which specifically inhibits C-fibers (27), was injected perineurally into the median nerve of rats with IMH (Figure 3A). The control group showed the development of hypertension following immobilization stress (QX-314 + Capsaicin, *n* = 5), which was attenuated by WAI stimulation at 52°C. The antihypertensive effect of WAI stimulation was almost completely blocked by pretreatment with QX314 and capsaicin (QX-314 + Capsaicin+WAI, two-way repeated ANOVA; group *F* (2,168) = 121.3, *p* < 0.0001; time *F* (13,168) = 94.21, *p* < 0.0001; interaction *F* (26,168) = 6.717, *p* < 0.0001; Figure 3B).

### 3.4. Involvement of peripheral TRPV1 receptors in the antihypertensive effect of WAI stimulation at PC-6 in rats with IMH

**Figure 4:**
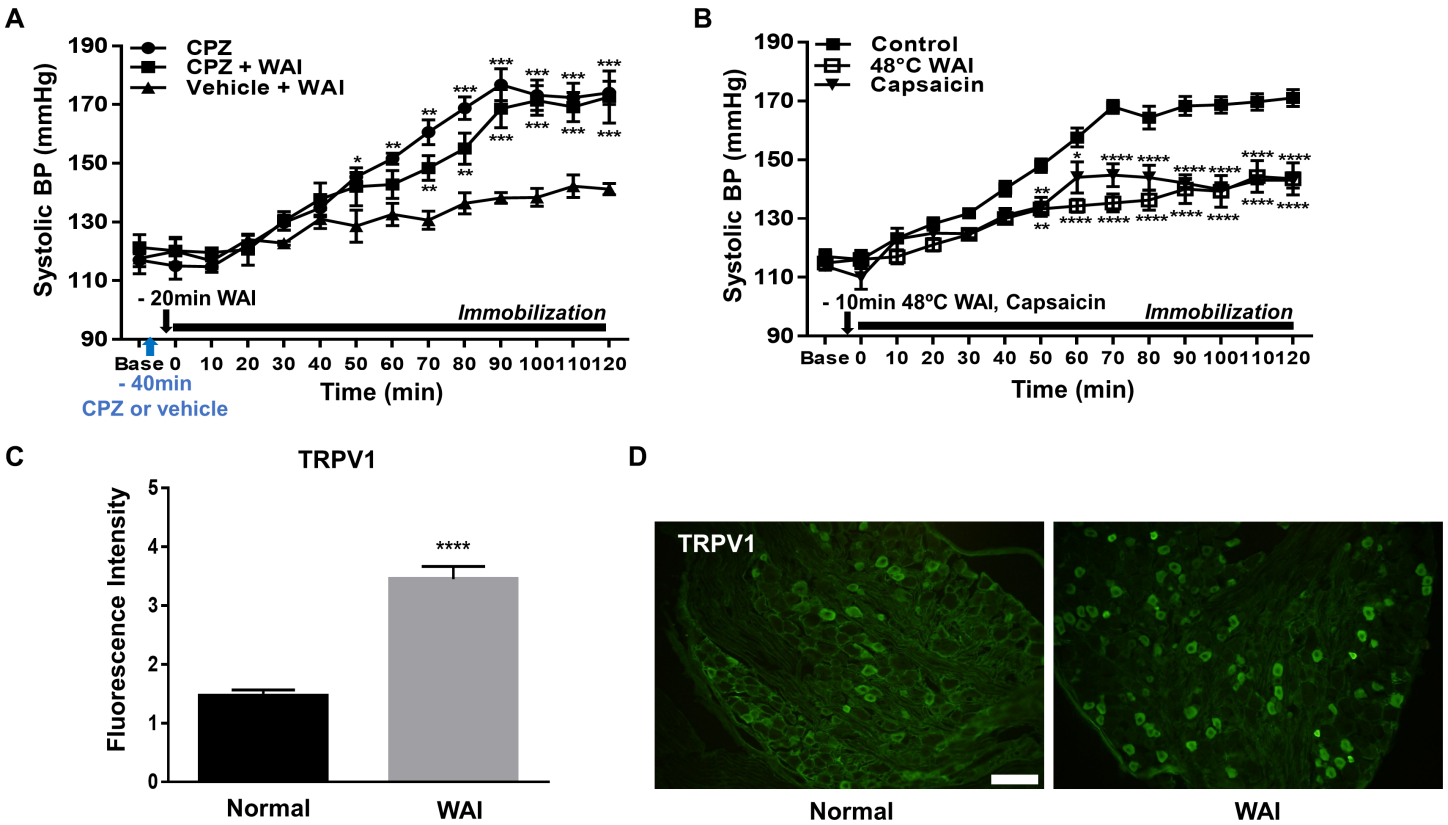
Effect of TRPV1 activation at PC-6 on systolic blood pressure in rats with immobilization stress-induced hypertension (IMH). **(A)** Effect of pretreatment with the TRPV1 receptor antagonist capsazepine (CPZ, *n* = 5) on PC-6 warm needle acupuncture instrument (WAI) stimulation-induced inhibition of hypertension. ^***^p < 0.001 vs. Vehicle + WAI (*n* = 5). CPZ, capsazepine into PC-6; CPZ, capsazepine into PC-6 prior to WAI stimulation at PC-6; Vehicle+WAI, vehicle injection into PC-6 prior to WAI stimulation. **(B)** Effect of the TRVP1 agonist capsaicin (*n* = 6) or 48°C application (*n* = 6) to the PC-6 acupoint on the development of hypertension in rats with IMH. ^****^p < 0.0001 vs. control (immobilization only, *n* = 6). **(C,D)** Immunohistochemistry for TRPV1 in DRGs following WAI stimulation at PC-6. Fluorescence intensity of immunopositive neurons in the normal (*n* = 7) and WAI (*n* = 7) groups **(C)**. ^****^p < 0.0001 vs. Normal. Representative epifluorescent images showing TRPV1 expression (green) in the dorsal root ganglia (DRGs) of the normal and WAI groups **(D)**. Scale bar: 100 μm.

To explore whether the antihypertensive effect of WAI stimulation at PC-6 is mediated through peripheral TRPV1 receptors, the TRPV1 antagonist capsazepine was injected into the PC-6 acupoint 40 min prior to the experiment. The development of hypertension was attenuated by WAI stimulation of PC-6 at 52°C in rats with IMH (Figure 4A; Vehicle+WAI), which was abolished by pretreatment with capsazepine (CPZ + WAI, two-way repeated ANOVA; group F (2,168) = 65.96, *p* < 0.0001; time F (13,168) = 53.13, *p* < 0.0001; interaction F (26,168) = 4.301, *p* < 0.0001.). On the other hand, when basal blood pressure before injection (Base) was compared with the value 40 min after injection (Time 0, immediately before immobilization), CPZ into PC6 itself did not affect the basal blood pressure. Also, CPZ injection itself did not affect the development of hypertension (Figure 4A). To further confirm whether the activation of TRPV1 receptors at PC-6 itself can produce antihypertensive effects, the TRPV1 agonist capsaicin was injected into PC-6 10 min prior to immobilization stress. The injection of capsaicin into PC-6 attenuated the development of IMH in rats. As previous studies showed activation of TRPV1 receptors at 48°C (31, 32), we tested whether WAI at 48°C could generate similar anti-hypertensive effects as capsaicin injection. The effect of capsaicin injection into PC6 on hypertension was similar to that of WAI stimulation at 48°C (two-way repeated ANOVA; group *F* (2,210) = 131.3, *p* < 0.0001; time *F* (13,210) = 65.10, *p* < 0.0001; interaction *F* (26,210) = 4.639, *p* < 0.0001.) (Figure 4B). To observe the activation of TRPV1 by WAI stimulation at 48°C, immunohistochemistry for TRPV1 in the DRGs of C6-T2 was carried out in another set of animals. The DRGs were removed 30 min after WAI stimulation at 48°C and analyzed. An almost 2-fold increase in TRPV1 was found in the WAI group compared with the normal group (t-test, *F* = 4.806, *p* < 0.0001; Figures 4C,D).

### 3.5. Activation of small afferent nerve fibers by WAI stimulation at PC-6

**Figure 5:**
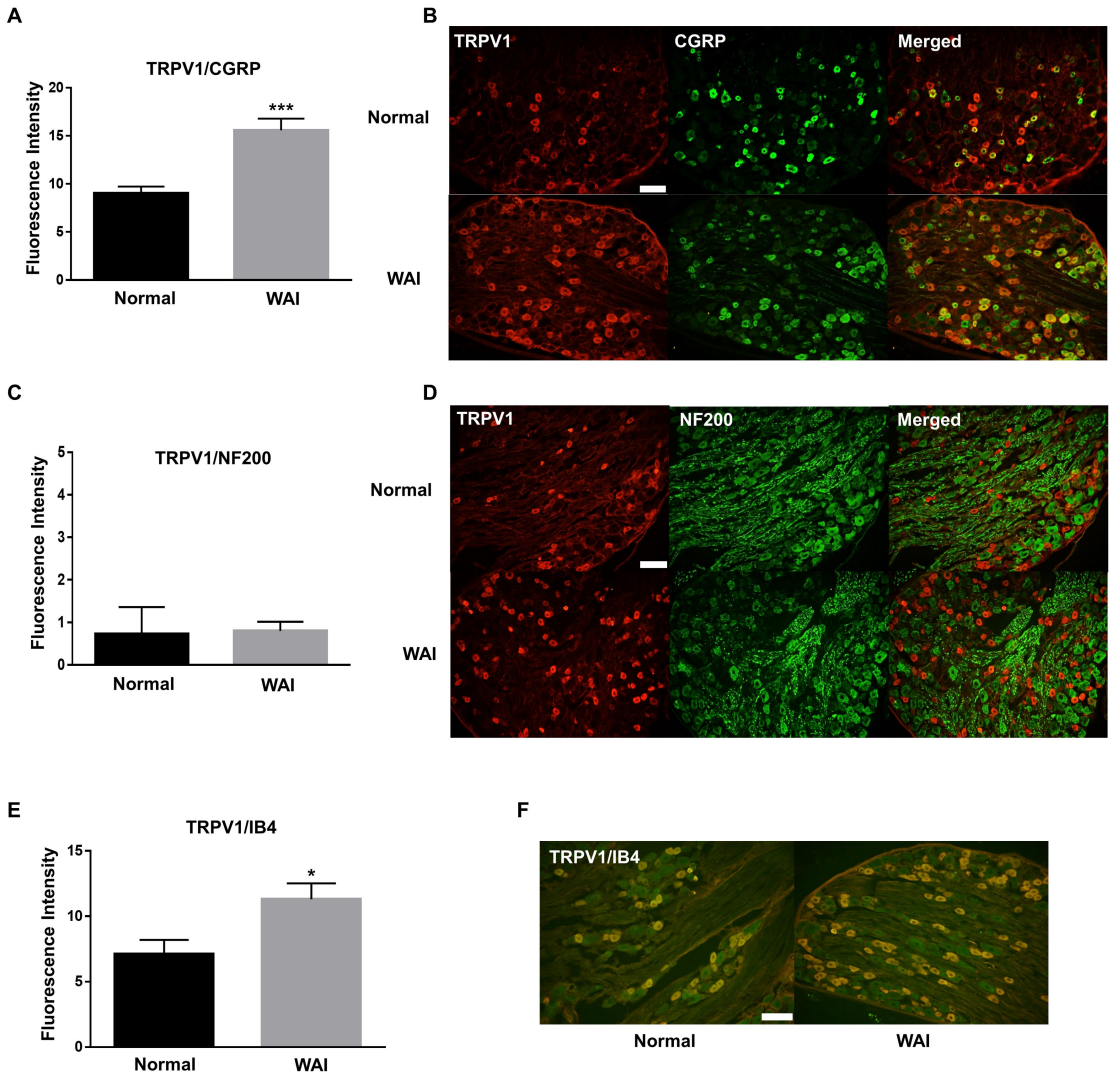
Immunohistochemistry for TRPV1 and/or CGRP, NF200 and IB4 in dorsal root ganglia (DRGs) following warm needle acupuncture instrument (WAI) stimulation at PC-6. **(A,B)** Fluorescence intensity and representative images of TRPV1 and CGRP double-stained DRG neurons (yellow) in the normal (*n* = 6) and WAI (*n* = 6) groups. ^***^ indicates a significance level of p < 0.001 vs. Normal. Immunofluorescent images for TRPV1 (red) and CGRP (green) **(C,D)** Fluorescence intensity and representative images of TRPV1- and NF200-positive DRG neurons in the normal (*n* = 6) and WAI (*n* = 6) groups. Immunofluorescent images for TRPV1 (red) and NF200 (green) **(E,F)** Fluorescence intensity and representative images of TRPV1- and IB4-positive DRG neurons in the normal (*n* = 6) and WAI (*n* = 6) groups. ^*^p < 0.05 vs. Normal. Double-stained cells (yellow) by TRPV1 (green)/IB4 (red). Scale bar: 100 μm.

To explore which subtypes of TRPV1-expressing DRG neurons were activated by WAI stimulation at PC-6, double-staining of TRPV1 and the neuronal markers CGRP (a peptidergic small neuronal marker), IB4 (a nonpeptidergic small neuronal marker) or NF200 (a marker for sensory myelinated fibers) was performed. The fluorescence intensity of TRPV1-immunoreactive neurons colocalized with CGRP was significantly higher in the WAI-stimulated group (*n* = 6) than in the normal group (Figures 5A,B) (*t*-test, *F* = 3.164, *p* < 0.001), and the number of TRPV1 neurons double-labeled with IB4 was significantly higher in the WAI group (*n* = 6) than in the normal group (Figures 5E,F) (*n* = 6, t-test, *F* = 1.249, *p* < 0.05). On the other hand, few neurons were double-labeled with NF200 and TRPV1 in the either the normal group or the WAI group (Figures 5C,D).

### 3.6. Inhibition of the PC-6-stimulation antihypertensive effect by ablating TRPV1 with RTX

**Figure 6:**
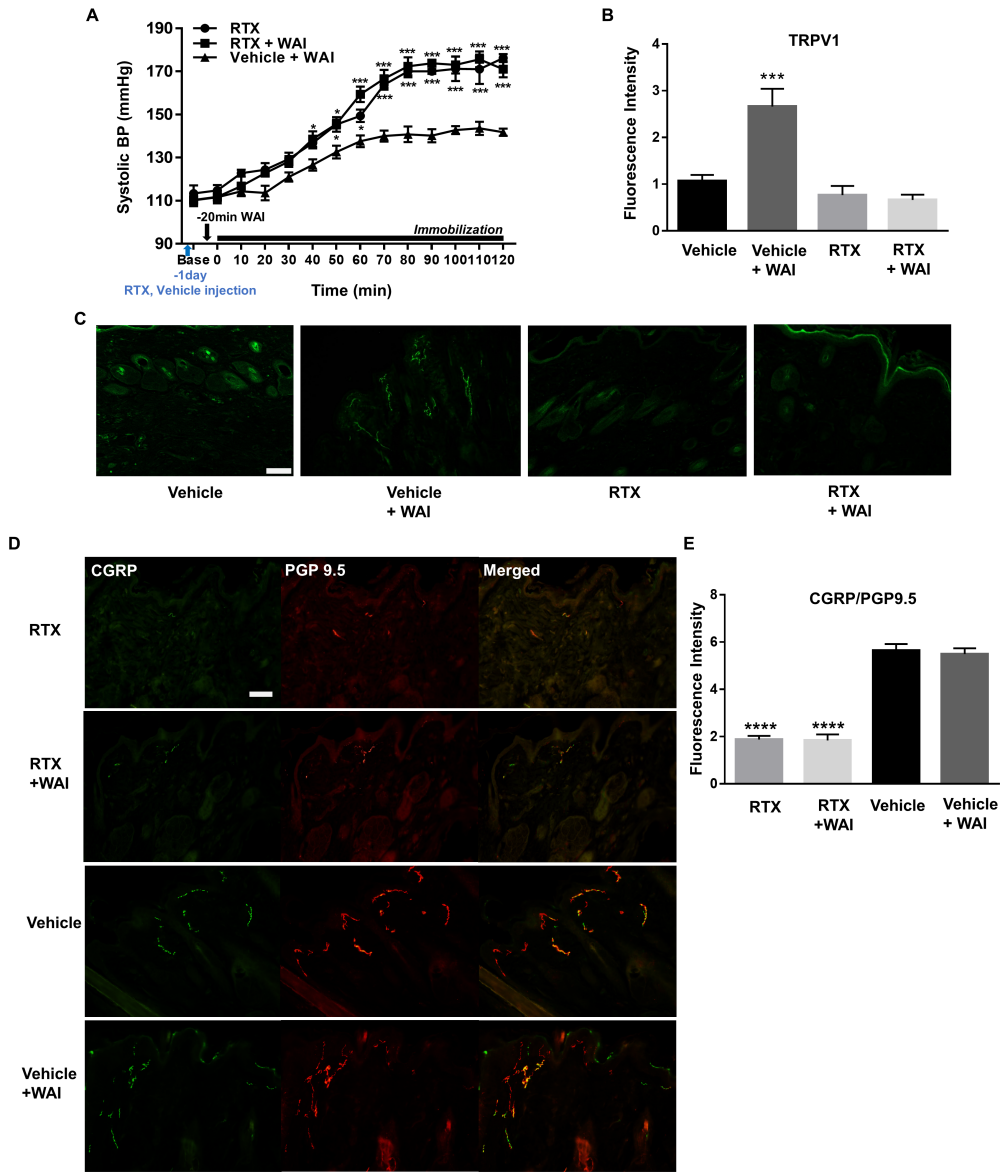
Effect of resiniferatoxin (RTX) preinjection on PC-6 warm needle acupuncture instrument (WAI) stimulation-induced inhibition of immobilization stress-induced hypertension (IMH) in rats. **(A)** Effect of RTX preinjection on PC-6 WAI stimulation-induced inhibition of IMH in rats. ^***^p < 0.0001 vs. Vehicle + WAI (n = 5). **(B,C)** TRPV1-positive neurons in the vehicle (*n* = 5), vehicle + WAI (*n* = 5), RTX (n = 5) and RTX + WAI (*n* = 5) groups. Representative images of TRPV1 neurons **(C)**. **(D,E)** Double staining of calcitonin gene-related peptide (CGRP, green) with the protein gene product 9.5 (PGP 9.5, red). Immunofluorescence intensity of CGRP/PGP 9.5-positive neurons (yellow) in the RTX (*n* = 5), RTX+ WAI (*n* = 5), Vehicle (*n* = 5) and Vehicle + WAI (*n* = 5) groups. ^****^p < 0.0001 vs. Vehicle. Scale bar = 100 μm.

To identify whether the ablation of peripheral TRPV1 channels at PC-6 inhibited the antihypertensive effect of WAI stimulation at PC-6, RTX, known to desensitize TRPV1 receptors (24), was injected into PC-6 in rats with IMH. While WAI stimulation of PC-6 at 52°C significantly attenuated the development of hypertension (Vehicle+WAI), compared to the control group (RTX), such effects were prevented by injecting RTX into PC-6 1 day before the experiment (Vehicle + WAI; two-way ANOVA; group *F* (2,168) = 128.5, *p* < 0.0001; time *F* (13,168) = 128.5, *p* < 0.0001; interaction *F* (26,168) = 5.104, *p* < 0.0001; Figure 6A). Immunohistochemistry for TRPV1 showed that the enhanced expression of TRPV1 induced by WAI stimulation at PC-6 was suppressed by pretreatment with RTX prior to WAI stimulation (one-way ANOVA; *F*=13.22, *p* < 0.0001; Figures 6B,C).

To investigate whether the nerve fibers of the skin were chemically ablated by RTX, we double-stained the nerve fibers with anti-PGP 9.5 antibody (a marker for intraepidermal general nerve fibers) and anti-CGRP antibody (a marker for peripheral peptidergic nerve fibers). The injection of RTX into PC-6 significantly decreased the fluorescence intensity of PGP9.5/CGRP compared to vehicle treatment (one-way ANOVA; *F* = 61.12, *p* < 0.0001; Figures 6D,E).

## 4. Discussion

We developed a novel temperature-controllable WAI mimicking traditional warm needling acupuncture (WA). WAI stimulation of PC-6 at 52°C significantly alleviated the development of immobilization stress-induced hypertension compared to that at 42°C. The antihypertensive effects of WAI stimulation at PC-6 were similar to those of traditional warm needle acupuncture. PC-6 pretreatment with the TRPV1 antagonist capsazepine prior to 52°C WAI stimulation blocked the antihypertensive effect of WAI stimulation at PC-6. Additionally, the activation of TRPV1 receptors at PC-6 by capsaicin or by 48°C WAI stimulation generated an antihypertensive effect. Immunohistochemical analysis showed that WAI stimulation increased TRPV1 expression in CGRP- or IB4-positive DRGs. The inhibition of peripheral TRPV1 by RTX injection into PC-6 prevented the antihypertensive effect of WAI stimulation. The present study found that warm needle acupuncture at PC-6 recruited peripheral TRPV1 receptors to attenuate the development of immobilization stress-induced hypertension in rats.

Warm needle acupuncture is known to be effective in the treatment of various diseases by combining the effects of thermal stimulation with those of manual acupuncture, which mechanically stimulates acupuncture points (11). Several studies have attempted to quantify the temperature of warm needle acupuncture to determine its mechanism of action (30, 33). However, it may not be easy to determine the optimal effective conditions in traditional warm needle acupuncture because the temperatures of warm needles vary depending on the size or weight of the moxa (30, 34). In a previous study, it was reported that high temperature stimulation at 47°C or 52°C produced a significant analgesic effect in mice with inflammation compared to low temperature stimulation at 37°C or 42°C (35). Moxibustion stimulation at 46°C significantly improved cardiac function by degranulating mast cells compared to 38°C stimulation (36). In the present study, PC-6 stimulation by a WAI produced an antihypertensive effect as well as traditional warm needling acupuncture, and WAI stimulation at 52°C more significantly inhibited the development of IMH in rats than WAI stimulation at 42°C. Similar to our findings, Gang et al. suggested that dry needle acupuncture at 44°C was effective in treating chronic myofascial pain syndrome in clinical trials (37), and in the CMPS rat model, dry needle acupuncture at 44°C significantly increased the mRNA expression of TRPV1, PKC, and IL-6 compared to stimulation at temperatures above 43°C (38). In addition, in HEK 293 cells heterologously expressing rat TRPV1, the cell current value increased from 44°C to 53°C in a temperature-dependent manner (39).

TRPV1 is a sodium/calcium ion channel that responds to noxious heat (>43°C), endogenous analgesic compounds and the vanilloid agonist capsaicin and is expressed in a subpopulation of DRG neurons (23). Previous studies have shown that EA stimulation increases TRPV1 activity and modulates sympathetic excitatory cardiovascular reflexes through central regulation of autonomic function (40). Our previous study also revealed that PC-6 stimulation by a TRPV1 agonist capsaicin patch reduced blood pressure in hypertensive participants (15). In the present study, the expression of TRPV1 in DRG neurons significantly increased following 48°C WAI stimulation at PC-6 compared to the normal group. The injection of capsazepine, a TRPV1 receptor antagonist, into acupoint PC-6 blocked the antihypertensive effect of WAI. Moreover, stimulation with 48°C WAI and a patch containing 1% capsaicin, a TRPV1 agonist, inhibited the development of hypertension in IMH rats. TRPV1 is an ion channel protein that opens at temperatures above 43°C and with capsaicin. Activated TRPV1 ion channels allow sodium and calcium ions to enter the cell, and the difference in ion concentration creates electrical signals that are transmitted to the central nervous system. It was reported that subepidermal nerve fibers expressing TRPV1 were higher in acupoint skin than in nonacupoint control skin and showed TRPV1 and C−/A-δ fiber colocalization. Moreover, acupuncture stimulation increased the expression of TRPV1 in nerve fibers (41, 42). Wu et al. found that TRPV1 was highly expressed at ST36 and participated in acupuncture-related analgesia, and capsaicin injection into ST36 replicated the analgesic effect of acupuncture (43). On the basis of these findings, we suggest that WAI stimulation at PC-6 increased the activity of peripheral TRPV1 to generate an antihypertensive effect in rats with IMH. Additionally, while a previous study reported that somatic pain-associated TRPV1 activation can produce sympathetic reflexes leading to an increase of blood pressure (44), the present study showed that focal activation of TRPV1 receptors at PC6 generated anti-hypertensive effects. In a previous study, application of electroacupuncture at PC6 points overlying the median nerve was most effective in patients with reflex-induced hypertension, while stimulation of points overlying the radial nerve or deep peroneal nerve was not effective or less effective (45, 46). Our previous study also showed that chemical stimulation of TRPV1 at PC6 over the median nerve reduced the systolic BP. The reductions in systolic BP are more profound after localized stimulation of the median nerve than after stimulation of the ulnar nerve site. It may suggest that the specific mediation of median nerve in the nerve stimulation-induced reduction of elevated BP and a critical contribution of TRPV1 receptors over median nerve to PC6-mediated BP reduction.

While electrical stimulation of the PC-6 acupoint can activate both A- and C-fibers of the median nerve (15, 47), it has been suggested that the effects of (EA) and transcutaneous electrical nerve stimulation (TENS) on cardiovascular diseases are mediated by C-fiber activation (46). For example, EA at PC-5 ~ 6 acupoints over the median nerve inhibits the reflex of the excitatory cardiovascular response induced by gastric distension (46), and the cardiovascular effect of EA is reduced by C-fiber blockade by neonatal treatment with capsaicin (48). Our previous study found that blocking A-fibers in hypertensive participants did not prevent the TENS-induced blood pressure-lowering effect, but stimulating C-fibers by capsaicin at the PC-6 acupoint produced acupuncture-like effects on hypertension (15). In the present study, we used a mixture of QX-314 and capsaicin to selectively block the C-fibers of the median nerve. Theoretically, with QX-314 and capsaicin coadministration, the pore of the TRPV1 channel is opened by capsaicin and becomes large enough to selectively transmit QX-314 or large molecules to nociceptive neurons, and the passed lidocaine derivative QX-314 selectively blocks the sodium channel (49), which induces excitatory inhibition of nociceptors and, as a result, selectively blocks the function of sensory neuron populations. In the present study, the antihypertensive effect of WAI stimulation at PC-6 was blocked by QX-314 and capsaicin pretreatment of the median nerve, suggesting that the mediation of C-fibers in the median nerve is required for WAI stimulation at PC-6 to produce antihypertensive effects. RTX, a TRPV1 agonist, leads to a sustained influx of sodium and calcium through the TRPV1 channel and leads to a loss of TRPV1-expressing DRG neurons or their fiber terminals through channel desensitization and calcium-induced cytotoxicity (50). In the present study, the administration of RTX into PC-6 inhibited TRPV1 activity and suppressed the antihypertensive effect of WAI stimulation at PC-6. TRPV1 channels play a major role in various disorders, such as pain, depression, stress, obesity and anxiety (51), and are known to be involved in various acupuncture effects. Previous studies have revealed that acupuncture stimulation increases TRPV1 activity in epidermal nerve fibers (41) and reduces body weight through TRPV1 activation in obese mouse models (52). On the basis of these findings, we suggest that WAI stimulation at PC-6 activates peripheral TRPV1 channels and C-fibers in the median nerve, thereby producing antihypertensive effects.

In conclusion, our present results showed that warm needle acupuncture produces an antihypertensive effect by activating C-fibers in the median nerve through peripheral TRPV1 activity in a rat model of immobilization stress-induced hypertension.

## Data availability statement

The raw data supporting the conclusions of this article will be made available by the authors, without undue reservation.

## Ethics statement

The animal study was reviewed and approved by Institutional Animal Care and Use Committee of the Korea Institute of Oriental Medicine.

## Author contributions

HK, YR, and SB contributed to conception and design of the study. SB, SC, SS, XJ, and S-YK conducted the experiments. K-HC and SJC performed the statistical analysis. SB, YR, and HK wrote the manuscript. All authors contributed to the article and approved the submitted version.

## Funding

This study was supported by the Korea Institute of Oriental Medicine, Korea (KSN1823212), and National Research Foundation of Korea (NRF) grant funded by the Korean government (MSIT) (No. 2020R1C1C1006534).

## Conflict of interest

The authors declare that the research was conducted in the absence of any commercial or financial relationships that could be construed as a potential conflict of interest.

## Publisher’s note

All claims expressed in this article are solely those of the authors and do not necessarily represent those of their affiliated organizations, or those of the publisher, the editors and the reviewers. Any product that may be evaluated in this article, or claim that may be made by its manufacturer, is not guaranteed or endorsed by the publisher.
